# Control of center of mass motion during walking correlates with gait and balance in people with incomplete spinal cord injury

**DOI:** 10.3389/fneur.2023.1146094

**Published:** 2023-05-30

**Authors:** Shamali Dusane, Anna Shafer, Wendy L. Ochs, Tara Cornwell, Heather Henderson, Kwang-Youn A. Kim, Keith E. Gordon

**Affiliations:** ^1^Department of Physical Therapy and Human Movement Sciences, Feinberg School of Medicine, Northwestern University, Chicago, IL, United States; ^2^Edward Hines Jr. VA Hospital, Hines, IL, United States; ^3^Department of Biomedical Engineering, Northwestern University, Evanston, IL, United States; ^4^Department of Preventive Medicine, Feinberg School of Medicine, Northwestern University, Chicago, IL, United States

**Keywords:** spinal cord injury, clinical balance measures, locomotion, lateral stability, stability

## Abstract

**Background:**

There is evidence that ambulatory people with incomplete spinal cord injury (iSCI) have an impaired ability to control lateral motion of their whole-body center of mass (COM) during walking. This impairment is believed to contribute to functional deficits in gait and balance, however that relationship is unclear. Thus, this cross-sectional study examines the relationship between the ability to control lateral COM motion during walking and functional measures of gait and balance in people with iSCI.

**Methods:**

We assessed the ability to control lateral COM motion during walking and conducted clinical gait and balance outcome measures on 20 ambulatory adults with chronic iSCI (C1-T10 injury, American Spinal Injury Association Impairment Scale C or D). To assess their ability to control lateral COM motion, participants performed three treadmill walking trials. During each trial, real-time lateral COM position and a target lane were projected on the treadmill. Participants were instructed to keep their lateral COM position within the lane. If successful, an automated control algorithm progressively reduced the lane width, making the task more challenging. If unsuccessful, the lane width increased. The adaptive lane width was designed to challenge each participant’s maximum capacity to control lateral COM motion during walking. To quantify control of lateral COM motion, we calculated lateral COM excursion during each gait cycle and then identified the minimum lateral COM excursion occurring during five consecutive gait cycles. Our clinical outcome measures were Berg Balance Scale (BBS), Timed Up and Go test (TUG), 10-Meter Walk Test (10MWT) and Functional Gait Assessment (FGA). We used a Spearman correlation analysis (*ρ*) to examine the relationship between minimum lateral COM excursion and clinical measures.

**Results:**

Minimum lateral COM excursion had significant moderate correlations with BBS (*ρ* = −0.54, *p* = 0.014), TUG (*ρ* = 0.59, *p* = 0.007), FGA (*ρ* = −0.59, *p* = 0.007), 10MWT-preferred (*ρ* = −0.59, *p* = 0.006) and 10MWT-fast (*ρ* = −0.68, *p* = 0.001).

**Conclusion:**

Control of lateral COM motion during walking is associated with a wide range of clinical gait and balance measures in people with iSCI. This finding suggests the ability to control lateral COM motion during walking could be a contributing factor to gait and balance in people with iSCI.

## Introduction

The requirements of the nervous system to actively control mediolateral motion of the whole-body center of mass (COM) during walking are substantial in comparison to fore-aft plane of motion that benefit from stabilizing body mechanics (1–3). In particular, the challenges of controlling this mediolateral motion are considerable during the single limb support phase of the gait cycle. Beginning at toe-off, the lateral velocity of the COM is relatively large and directed towards the stance limb. To maintain a straight-ahead walking trajectory, this lateral velocity must be reduced to zero (typically occurring around midstance as the COM position reaches its most lateral excursion and is positioned above the supporting limb), and then redirected towards the midline. Failure to arrest the lateral momentum of the COM will result in motion beyond the lateral base of support border (determined by the stance limb medio-lateral foot-placement). COM travelling beyond the lateral base of support border will require a corrective step(s) to restore the desired forward walking trajectory and prevent a fall. External moments acting in the frontal-plane about the ankle joint of the stance limb work collectively to arrest and then redirect the COM lateral velocity (3). The nervous system will use a combination of anticipatory and reactive control mechanisms to modulate these moments. Thus, the capacity to control lateral COM motion is likely a fundamental component of effective forward walking.

Growing evidence suggests that ambulatory people with incomplete spinal cord injury (iSCI) have considerable challenges controlling their lateral COM motions during walking. This includes difficulty arresting lateral motion after a walking maneuver (4, 5), impaired mediolateral foot placement (6), limited ability to increase lateral margins of stability following a perturbation (7), and sizeable metabolic energy cost to stabilize lateral motion during walking (8). Perhaps as a consequence of these challenges, ambulatory people with iSCI who walk without assistive devices demonstrate a substantially larger lateral COM excursion during walking at preferred and fast speeds when compared to age matched adults without iSCI (5). As such, many of the cautious gait patterns observed in people with iSCI [e.g., slower walking speeds, wider steps, shorter steps, more time in double support (6, 9–12)] have been suggested as compensatory mechanisms that proactively aid in COM control during walking (9, 10, 12).

Studies in populations without neurologic injuries have found that the control of mediolateral COM motion is critical for maintaining dynamic balance (13) and creating walking stability during directional changes (14). Almost all activities of daily living involve directional changes or turning maneuvers that require reorientation of the body in the anticipated direction of travel (15). Therefore, the ability to control lateral COM motion during walking may be a skill fundamental to functional gait and balance. If this relationship is supported, interventions that directly aim to improve the ability to control lateral COM motion may translate to improvements in gait and balance. The first step is to identify if the ability to control lateral COM motion during walking is related to functional gait and balance in people with iSCI.

The ability to control lateral COM motion during walking could be related to several functional measures of gait and balance. We selected four clinical outcome measures, the Functional Gait Assessment (FGA), the Timed Up and Go test (TUG), the 10-meter walk test (10MWT), and the Berg Balance Scale (BBS), that collectively would provide insights into the relationship between the ability to control lateral COM motion during walking and walking balance, walking speed, and postural balance, respectively. People with iSCI have impaired abilities to control lateral motion during walking (4–7), which may reduce functional walking balance on tasks requiring turns and change of gait speed. In the current study, we used the TUG (16–18) and the FGA (19–21) to examine walking balance. Both these tests include turns and changes in walking speed. People with iSCI often select cautious gait patterns, including walking at slower speeds (9, 10, 12), that reduces COM velocity. Slower COM motions are believed to enhance gait stability by decreasing perturbation intensities (9, 12). Thus, it seems likely that there could also be a relationship between the ability to control COM motion during walking and walking speed. We conducted the 10MWT, a widely used and recommended measure to assess gait speed (22–25), and analyzed relationship of preferred and fast 10MWT speeds with the ability to control lateral COM motion. Finally, we used the BBS to evaluate postural balance. The BBS has strong correlations with walking ability in people with iSCI (26–29), suggesting that similar mechanisms may be responsible for controlling COM dynamics during walking and standing. In the current study, we used the BBS to examine if the ability to control lateral COM motion during walking is related to postural balance. While these measures provide an overview of functional gait and balance, it should be noted that they do not provide a direct method to assess the ability to control lateral COM motion during walking.

We are not aware of any studies that have previously aimed to quantify people’s capacity to control (minimize) lateral COM motion during walking, however two studies have successfully used visual feedback to encourage people without neurologic injuries to minimize vertical oscillations of the COM during walking (30, 31). Based on these prior studies, to quantify the ability to control lateral COM motion during walking, we developed a treadmill-based assessment that used visual feedback to encourage people to minimize lateral COM motion during forward walking. During this assessment, a target walking lane and the real-time mediolateral position of the participant’s COM are projected on the walking surface of an oversized treadmill. Participants are instructed to maintain their mediolateral COM position within the projected lane during walking. If participants maintain their COM within the target lane, the lane width is progressively decreased, increasing the challenge of the walking task. This external visual feedback encouraged participants to try their best to control their COM motion during forward walking. To quantify the participants’ capacity to control their lateral COM motion, we performed a post-hoc kinematic analysis to identify the minimum lateral COM excursion occurring during five consecutive gait cycles.

The purpose of this study was to evaluate the relationship between the ability to control lateral COM motion during walking and validated clinical gait and balance measures commonly used to assess ambulatory people with iSCI. We hypothesized that the ability to control lateral COM motion during walking would correlate with clinical gait and balance outcome measure scores.

## Materials and methods

### Participants

Twenty adults with chronic incomplete spinal cord injury participated in this cross-sectional study. All participants had spinal cord injuries between C1-T10 and were classified as C or D on the American Spinal Injury Association Impairment Scale (AIS). Our inclusion criteria were the following: age between 18 to 80 years, more than 6 months post-incomplete spinal cord injury, medically stable, and able to ambulate 10 m without physical assistance or use of assistive devices. Our exclusion criteria were the following: excessive spasticity in the lower limbs (>3 on the Modified Ashworth Scale), unable to tolerate 10 min of standing, presence of severe cardiovascular and pulmonary disease, unhealed decubiti or other skin compromise, history of recurrent fractures or known orthopedic problems in the lower extremities, concomitant central or peripheral neurological injury, unable to provide informed consent due to cognitive impairments, enrolled in concurrent physical therapy or research involving locomotor training, and use of braces/orthotics crossing the knee joint. The criteria for participation in this study, in particular the ability to walk without assistive devices, will inherently result in a participant sample that is considered high functioning. As our objective was to assess the relationship between the ability of the individual to control lateral COM motion and functional gait and balance, we restricted the use of assistive devices, because devices like canes and walkers will aid in the control of COM motion. We believe the relationship between the ability to control COM motion and functional gait and balance will be greatest in those who walk without assistive devices. Thus, in the current study we limited our study population to individuals who were able to walk without assistive devices.

This study was conducted at the Human Agility Laboratory, Physical Therapy and Human Movement Sciences, Northwestern University Feinberg School of Medicine. The study protocol was approved by the Institutional Review Boards at Northwestern University and the Edward Hines Jr. Veterans Affairs Hospital. All participants provided informed written consent prior to enrollment in the study.

### Experimental setup

To assess participants’ ability to control their lateral COM motion during walking, we recorded kinematic data as participants walked on an oversized treadmill, walking surface 2.6 × 1.4 m, (TuffTread, Willis, TX) while receiving visual feedback about their lateral COM position. For safety, participants wore a trunk harness attached to passive overhead support that did not provide bodyweight support (Aretech, Ashburn, VA). The harness straps were adjusted to allow participants unrestricted lateral travel across the treadmill. During treadmill walking, participants were not allowed to use any assistive devices (canes, walkers, handrails) except for any passive ankle-foot orthoses they would typically wear during community ambulation. In the event of a loss of balance, a physical therapist providing standby assistance would give manual support as necessary to allow the participant to regain balance and continue walking. During the treadmill-based assessment of participants’ ability to control their lateral COM motion, participants were not allowed to use any assistive devices (canes, walkers, handrails) except for any passive ankle-foot orthoses. In addition, we only analyzed walking periods when no manual assistance was provided.

During treadmill walking, we used a 12-camera motion capture system (Qualisys, Gothenburg Sweden) to collect 3D coordinates of 19 reflective markers placed on the pelvis and lower limbs at 100 Hz. Markers were placed at the following locations: S2 vertebrae and bilaterally on each sacroiliac joint, greater trochanter, anterior superior iliac spine, highest point of the iliac crest, lateral malleolus, calcaneus, and the 2nd, 3rd, and 5th metatarsals.

We evaluated participant’s capacity to control their lateral COM motion during three treadmill walking trials that were each 21 m in length. During the trials participants were given visual feedback of their lateral COM position to challenge them to minimize their lateral motion during walking (Supplementary video S1). Specifically, their real-time mediolateral COM position was represented by a white line projected along the length of the treadmill surface using a short throw projector mounted on the wall alongside the treadmill (Hitachi, Tokyo, Japan). The lateral COM position was calculated using real-time 3D locations of the pelvis markers that were streamed to a custom-programmed control algorithm (LabVIEW, National Instruments, Austin, TX). The control algorithm calculated mediolateral COM position as the midpoint between the two greater trochanter markers (32) and transformed the data into the treadmill coordinate system for display.

**Figure 1:**
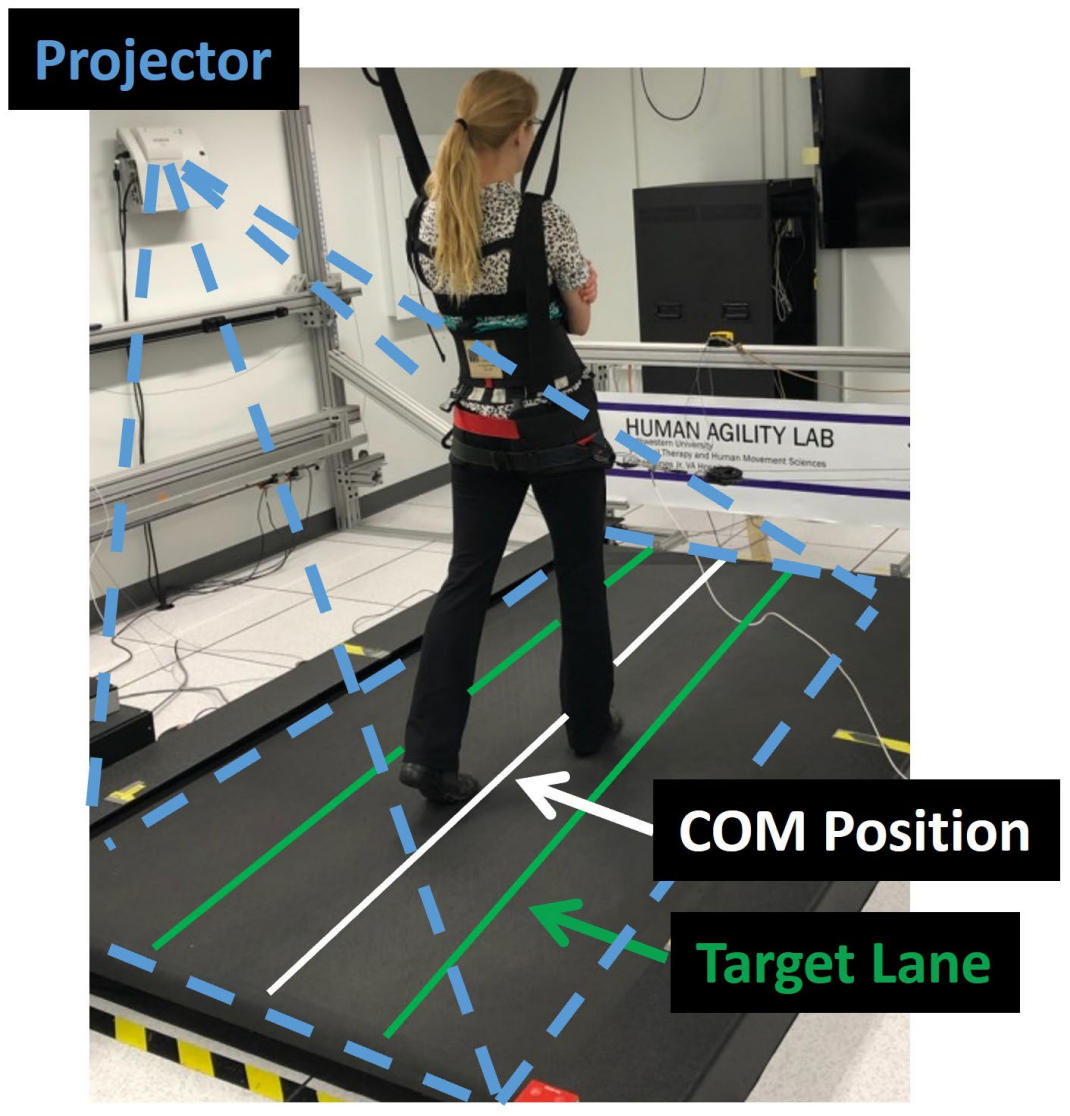
Experimental set-up. The laboratory balance assessment is performed three times on an oversized treadmill to test an individual’s lateral center of mass (COM) control. The real-time mediolateral COM position of the participant is projected on the treadmill through a white line. Participants are instructed to walk at their preferred treadmill speed and to do their best to keep the white line within the green target lane. If successful, the target lane width is progressively decreased. If not, the area outside of the target lane (to either the left or right) changes to red, providing an immediate visual cue that the participant has made an error and should try to return the white line to the projected increased lane.

Additionally, lateral boundary targets for COM position (“target lane”) were projected on the treadmill (Figure 1). To maximally challenge participants to minimize their lateral COM motion during walking, the control algorithm systematically adjusted the width of the target lane based on how successful the participant was at maintaining their lateral COM position within the green target lane (Figure 1). During walking, if the COM moved outside the target lane, that area outside the target turned red to provide an immediate visual cue to return to the green target lane. At the beginning of the first walking trial, the initial lane width was set to 200 mm. Our prior research found that people with iSCI who were able to ambulate with no assistive devices or manual assistance had an average lateral COM excursion per stride of 80 mm (5). Thus, we selected a starting width of 200 mm with the goal that the lane would be sufficiently wide that all participants in this study would be successful at the start of the trial. Once the assessment began, the control algorithm made a 10 mm step change in the lane width based on the following logic: if the participant maintained their lateral COM position in the lane for 1.5 consecutive meters of forward walking, the lane width was decreased by 10 mm. If the participant walked for 3 meters without maintaining COM position within the lane for at least 1.5 consecutive meters, the lane width increased by 10 mm. We selected to make iterative changes in the lane width of 10 mm based on two competing factors. First, we wanted the iterative changes to be large enough that the task would converge on an optimally challenging width for each participant by the participant’s second 21 m trial. Second, we wanted the iterative changes to be small enough that the task would adjust gradually with sufficient resolution to capture each participant’s optimal ability. Based on these criteria and extensive pilot testing we selected 10 mm iterative changes. A minimum lane width was set at 5 mm based on the resolution of the lines projected on the treadmill. Consecutive walking assessments started at the lane width achieved at the end of the previous trial (e.g., the starting lane width for the second assessment was equal to the ending lane width of the first assessment). The algorithm thresholds were established in pilot testing prior to the current study to minimize walking time yet converge on the smallest lane width participants could maintain.

We selected 21 m as the distance for each individual treadmill trial length for two reasons. First, we wanted to select a distance that our participants would likely be able to walk continuously without requiring a rest break. Given that the inclusion criteria for the study was the ability to walk 10 m without assistance, we felt that 21 m should be accomplishable for most participants. Second, we wanted to select a distance that was equally divisible by both 1.5 m and 3 m, the distances the algorithm used to evaluate performance and then iteratively update the width of the feedback lane.

We used a fixed walking distance for each trial (21 m) and updated the lane width at fixed intervals of distance traveled (either 1.5 m or 3.0 m) that was consistent across all participants. The purpose of the visual feedback was specifically to encourage participants to reduce their COM excursion. We updated the lane based on the distance traveled to ensure that the number of times the target lane width updated within a trial was consistent across all participants independent of how fast they walked or their stride length.

### Clinical outcome measures

*Timed Up and Go test (TUG):* The TUG is a valid and reliable measure for functional mobility, balance, and fall-risk in people with iSCI (16, 17). It is a widely used and recommended screening tool for prediction of fall-risk (33–35). The participant is asked to rise from the chair, walk 3 meters safely, turn around, walk back, and sit down on the chair. The TUG score is the time recorded from when participants rise from the chair until they sit down again.

*Functional Gait Assessment (FGA):* The FGA is a valid and reliable test that measures balance and gait functions in people with iSCI (20, 21). It is a modification of the Dynamic Gait Index (19) consisting of 10 items scored on a four-point ordinal scale ranging from 0 to 3, such that 0 indicates severe impairment and 3 indicates normal/no impairment with a total score of 30. The clinical practice guideline recommends usage of FGA as a core outcome for patients with neurological conditions (36).

*Berg Balance Scale (BBS):* The BBS is a 14-item scale, widely used, valid, and reliable measure for balance assessment of people with iSCI during predetermined tasks performed in daily living (27–29). Each item is scored on a five-point ordinal scale ranging from 0 to 4, such that 0 signifies the lowest level of function and 4 signifies the highest level of function. The maximum total score is 56, with higher total scores indicating better balance (37). The BBS has also been useful as a screening tool to predict risk of falls beyond cut-off scores (38–40).

*10-Meter Walk Test (10MWT):* The 10MWT has been found to be a valid and reliable test to measure overground walking speed, both preferred and fast, in people with iSCI (17, 41–43). It is a recommended measure for assessment of gait speed among neurological populations (24, 25).

For the clinical measures assessment, participants were allowed to use assistive devices such as cane, rolling walker and/or ankle foot orthosis they would typically wear during community ambulation.

### Protocol

Participants in the current study were part of a larger clinical trial investigating a high intensity gait training intervention. As such, all participants first underwent an extensive clinical assessment prior to future enrollment in the intervention (any participation in the gait training intervention occurred 1-week after completing all testing described in the current study). A licensed physical therapist collected demographic information (age, gender, date of birth), date of spinal cord injury, level of spinal cord injury, cause of spinal cord injury, current, and past medical history, current medications (to screen for use of beta blockers which could potentially affect heart rate during the gait training intervention and to evaluate if any medications could have resulted in balance deficits as a side effect), current ambulatory ability in the home and community (including the use of any assistive devices), and self-reported number of falls in the past year. The physical therapist then collected four clinical outcome measures: BBS, TUG, FGA, and 10MWT at preferred and fast speeds.

Next, participants performed the treadmill walking portion of the experiment that was used to assess their ability to control their lateral COM motion. The participant’s preferred treadmill walking speed was identified through a staircase method of increasing and decreasing the treadmill speed until the participant’s desired speed is confirmed through verbal feedback. Participants were given several minutes to accommodate to walking on the treadmill at this preferred speed.

With the treadmill stopped, participants were then given detailed instructions about the assessment to be performed. The projector used to display the participant’s real-time lateral COM position and a target lane was turned on. Participants were instructed to make some small movements to their left and right so that they understood that the side-to-side movement of their body controlled the position of the white line being projected on the treadmill. Participants were asked to perform three walking trials of 21 m each. Participants were instructed to do their best to maintain the white line representing the midline of their body within the target lane. They were also told that if they were successful, the width of the target lane would be progressively reduced. Once the participant understood the instructions, the treadmill was started, and participants were given time to reach steady-state before the assessment began. At the end of the 21 m assessment, the treadmill was stopped, and participants were given time to rest as needed. Then two more 21 m assessments, separated by a rest break, were performed. Consecutive walking assessments started at the lane width achieved at the end of the previous trial (e.g., the starting lane width for the second assessment was equal to the ending lane width of the first assessment).

### Data analysis and processing

Data from all treadmill walking assessments were examined and used to estimate participants’ ability to control their lateral COM motion during treadmill walking. Kinematic marker data was processed using Visual3D (C-Motion, Germantown, MD) and a custom MATLAB (Mathworks, Natick, MA) program. Marker data was gap-filled and low-pass filtered (Butterworth, 6 Hz cut-off frequency). Time of initial foot contact (IC) and toe-off (TO) events were identified for each step based on maximum and minimum fore-aft positions of the calcaneus and 2nd metatarsal markers, respectively. Mediolateral COM position was calculated in Visual3D as the center of the Visual3D model’s pelvis. We then calculated the lateral COM excursion for each gait cycle. Finally, from all gait cycles, we identified the five consecutive gait cycles that produced the smallest average lateral COM excursion. This value, the minimum lateral COM excursion over five consecutive gait cycles, was used to represent each participant’s ability to control their lateral COM motion during forward walking.

### Statistical analysis

Descriptive variables, scores of clinical outcome measures, and minimum lateral COM excursion were reported as mean [standard deviation (SD)]. The Shapiro–Wilk test for normality was used to confirm the assumption of normality of clinical balance measures and minimum lateral COM excursion. To evaluate the relationship between the ability to control lateral center of mass motion during walking and clinical outcome measures, a Spearman correlation analysis was performed between minimum lateral COM excursion and the following clinical outcome measures: TUG, FGA, BBS and preferred and fast 10MWT. Spearman’s correlation coefficients (*ρ*) used for all the measures was interpreted as follows: >0.70 as strong, 0.50–0.70 as moderate, 0.30–0.50 as weak (44). A valuable property of the Spearman coefficient is that it is relatively robust against outliers because it quantifies a strictly monotonic relationship between two variables (45). All statistical analyses were performed using SPSS (Version 24, SPSS Inc., Chicago, IL, United States) with *α* = 0.05. A Bonferroni correction was applied to account for multiple correlations with level of significance of 0.01.

## Results

Twenty people with iSCI participated in our study (mean age of 52.9 ± 18.2 years). Participant characteristics are reported in Table 1. Participants mean scores of clinical and laboratory outcomes are reported in Table 2. All participants were able to successfully complete the walking assessments. The participants found the laboratory-based assessment to be challenging yet engaging and enjoyable. Participants were able to use the visual feedback to make changes in the way they walked.

**Table 1:** Participant characteristics.

Demographics (*n* = 20)	Results
Age in years, mean (SD)	52.9 (18.06)
Gender	
Male	*n* = 14; 70%
Female	*n* = 6; 30%
Height in meters, mean (SD)	1.73 (0.07)
Years post SCI, mean (SD)	7.95 (9.06)
Level of injury, number, and percent of participants	
Cervical	*n* = 13; 65%
Thoracic	*n* = 7; 35%
Mechanism of injury, number, and percent of participants	
Traumatic	*n* = 15; 75%
Non-traumatic	*n* = 5; 25%
Self-selected Walking Index for Spinal Cord Injury (WISCI II), mean (SD)	17.65 (2.74)
Lower Extremity Motor Score (LEMS), mean (SD)	43.85 (4.69)
Walking device, number and percent of participants	
Rolling walker	*n* = 1; 5%
Cane	*n* = 10; 50%
None	*n* = 9; 45%
Brace usage, number and percent of participants	
Using brace	*n* = 4; 20%
Not using brace	*n* = 16; 80%
Falls, number and percent of participants	
No falls	*n* = 7; 35%
1 fall	*n* = 4; 20%
2 or more falls	*n* = 9; 45%

**Table 2:** Clinical and laboratory outcome measures.

Outcome measure	Mean score (SD)
Timed Up and Go test (TUG) (seconds)	18.86 (16.13)
Functional Gait Assessment (FGA)	18.40 (7.26)
Berg Balance Scale (BBS)	49.55 (6.19)
10meter walk test (10MWT)	
Preferred overground walking speed (meter/s)	0.84 (0.33)
Fast overground walking speed (meter/s)	1.13 (0.43)
Minimum lateral center of mass (COM) excursion (mm)	54.67 (30.45)

**Figure 2:**
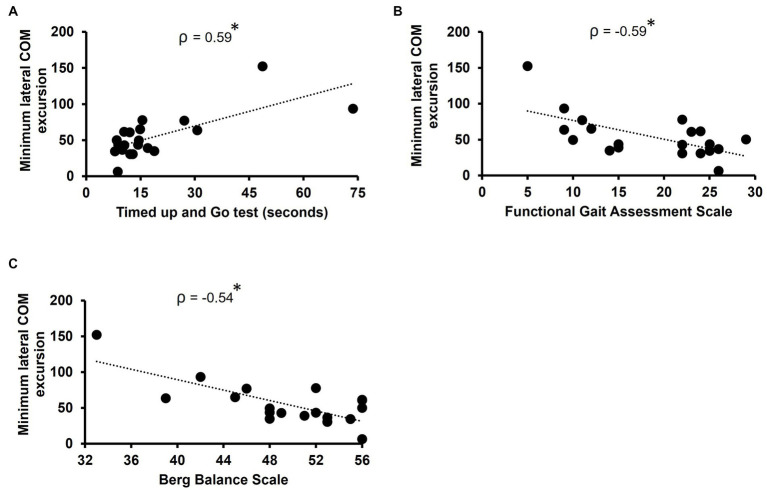
Scatterplots of minimum lateral COM excursion with **(A)** Timed Up and Go test, **(B)** Functional gait assessment scale, and **(C)** Berg balance scale. Each circle represents one participant. *p < 0.01.

**Figure 3:**
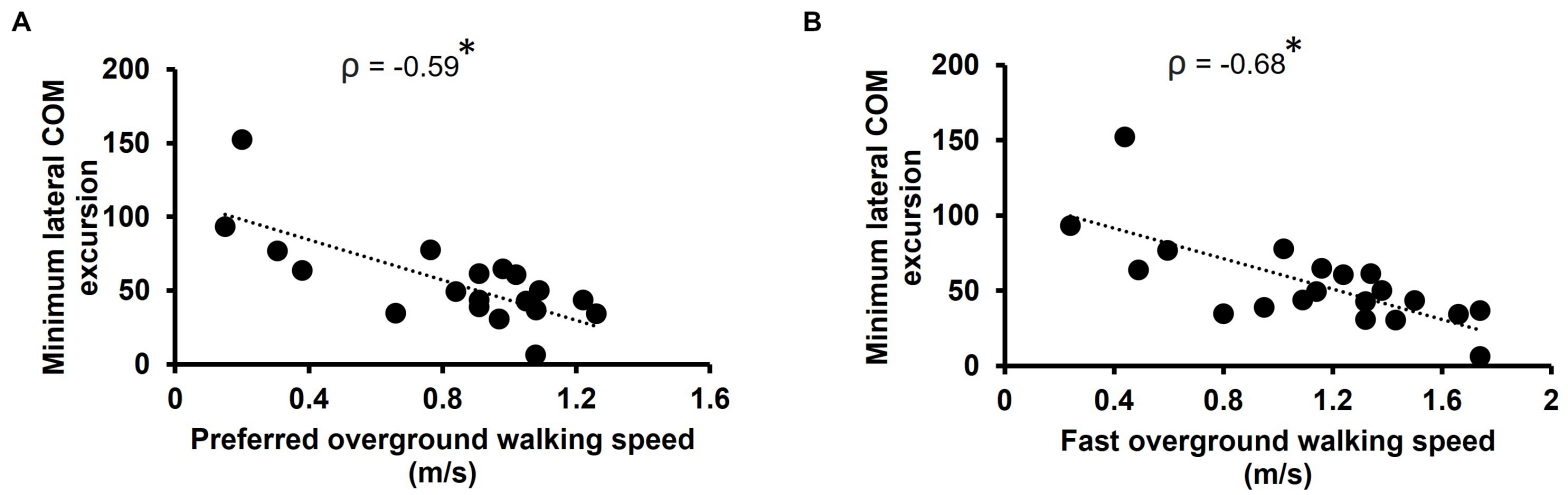
Scatterplots of minimum lateral COM excursion with **(A)** preferred overground walking speed as measured by the 10MWT and **(B)** fast overground walking speed as measured by the 10MWT. Each circle represents one participant. *p < 0.01.

Minimum lateral COM excursion significantly correlated with all clinical outcome measures we examined. There was a moderate, positive correlation between minimum lateral COM excursion and TUG time (*ρ* = 0.59, *p* = 0.007) (Figure 2A). There was a moderate, negative correlation between minimum lateral COM excursion and FGA score (*ρ* = −0.59, *p* = 0.007) (Figure 2B) and BBS score (*ρ* = −0.54, *p* = 0.014) (Figure 2C). The minimum lateral COM excursion had a moderate, negative correlation with fast 10MWT speed (*ρ* = −0.68; *p* = 0.001) (Figure 3A) and with preferred 10MWT speed (*ρ* = −0.59; *p* = 0.006) (Figure 3B).

## Discussion

In support of our hypothesis, we found that for ambulatory adults with iSCI, the ability to control their lateral COM motion during walking moderately correlated with clinical outcome measures related to walking balance, walking speed, and postural balance.

The ability to control lateral COM motion during walking was found to be moderately correlated with our two clinical measures of walking balance, the TUG and FGA. Smaller minimum lateral COM excursions were associated with shorter TUG times and higher scores on the FGA, both indicating greater walking ability. A moderate correlation indicated that both (clinical measure and lateral COM excursion) measure a similar construct, i.e., walking balance. Both the TUG and FGA involve walking and maneuvering with a pivot turn that challenges functional walking balance. Specifically, the FGA provides a range of gait activities (change in gait speed, head turns, narrow base of support walking) that require complex stabilizing strategies and postural adjustments. Whereas the TUG requires motor planning and capacity to anticipate transitioning from one motor task to another in a particular sequence (46). Due to their sensorimotor deficits, reduced muscle strength, impaired proprioception and deficits in balance and coordination, people with iSCI have difficulty with safely performing complex maneuvers during walking. These impairments challenge dynamic balance and their ability to make anticipatory changes during walking. Our finding that individuals with the poorest ability to control their lateral COM excursion also had the lowest scores of FGA and took the longest time to perform TUG is built on previous findings indicating that people with iSCI have impaired control of lateral motion during walking (4–7).

Similar to the TUG and FGA, minimum lateral COM excursion showed a moderate, negative correlation with BBS score. Specifically, individuals with greater BBS scores, which indicate greater postural control and balance, demonstrated a better ability to control their lateral COM excursion during walking. The BBS is often referred to as a “gold standard” because it is one of the most widely used, valid, and reliable clinical measures for assessing balance and postural control (28, 29, 38, 47, 48). Since scoring for BBS is based on how well the participant performs a series of balance challenging tasks that are performed in daily life, it is a good indicator of functional balance (including static and dynamic) and is used for assessment of fall-risk in several populations (49–52). Previous studies examining the relationship between balance measured by BBS and walking ability in neurological population found that BBS score is a strong predictor of walking ability (home and community ambulation) among people with stroke (53–55). BBS score is also found to correlate with walking performance in people with SCI (26) demonstrating a strong relationship between walking function and balance. Our results support this relationship such that people with iSCI who had the best ability to control their lateral COM excursion during walking had the highest scores on the BBS. Because our study participants were relatively high functioning there was a possibility that the BBS may be susceptible to a ceiling effect. In our study, 4 of our 20 participants scored a 56, the highest score on the BBS. Thus, majority of our participants did not experience a ceiling effect. Despite the potential for ceiling effects, we felt it was important to include this measure of balance in the study because of its extensive use and its recommendation by the Spinal Cord Injury EDGE Task Force as one of the measure of balance (56).

Our results also demonstrated a correlation between better lateral COM control during walking and walking speed. This relationship was moderate for both preferred and fast walking speed. These results could be attributed to an association between changes in COM excursion with walking speed. Studies in healthy adults reported that lateral COM excursion decreases with increase in walking speed (57) and restrictions to COM excursion lead to increased walking speeds in order to ensure dynamic stability (58). Thus, the ability to control lateral COM excursion could provide stability that enables individuals to walk faster.

We developed a novel task to assess participants’ maximum capacity to control their lateral COM motion during forward walking. We provided participants with visual feedback by projecting a target lane on the treadmill to encourage them to try and minimize their lateral motion during walking. We then assessed their best performance during five consecutive gait cycles occurring at any time during three trials of the 21 m walking task. We used five consecutive gait cycles to evaluate performance as this brief, but sustained period is likely to have minimized the likelihood that this top performance was by chance. While this task was engaging and challenging for participants, the task itself may have some limitations as a method of evaluating the ability to control lateral COM motion. For example, to receive visual feedback, participants had to look down at the treadmill walking surface. It is possible that this requirement may have affected participants’ normal walking pattern. However, in developing this protocol we have found that participants with iSCI strongly prefer visual feedback to be provided on the treadmill rather than on a monitor mounted at eye-level in front of the treadmill that require participants to “look up.” This is likely because people with iSCI often use vision for foot placement during walking (59, 60). In addition, this task is only able to evaluate one component of walking – maximum ability to control lateral COM motion during forward walking. However, as the correlations between the control of lateral COM motion and our functional gait and balance measures demonstrated moderate strength of association, it is clear that there are other underlying skills that will contribute to performance. For example, in the current trial we evaluated performance during consecutive gait cycles during treadmill walking in a relatively consistent environment. During real-world walking and balance tasks, people may be required to make regular step-to-step changes to continually adjust their foot placement in order to react to small changes in their COM state (position and velocity). Indeed, in our prior work we have found that increases in step-to-step variability of lateral foot placement when walking at fast speeds may be a method that people with iSCI use to maintain stability of lateral COM dynamics during walking (5).

Our findings have important clinical implications for balance assessment and training. Our study indicates that the ability to control lateral COM motion during walking is closely related to several functional measures of gait and balance in people with iSCI. We believe this relationship may be because control of lateral COM motion during walking and the gait and balance measures we examined are all dependent on the capacity of the nervous system to both anticipate and react to ongoing COM dynamics. People with iSCI have an impaired ability to anticipate COM dynamics due to sensory (61) and motor (7) dysfunction. Additionally, research has found deficits in reactive balance responses used to control lateral COM motion in this population (62). Thus, limitations in the ability to accurately anticipate and react to ongoing COM dynamics may reduce their ability to control lateral COM motion during walking and to perform functional measures of gait and balance that require these fundamental skills. This relationship could motivate clinical interventions that directly target the ability to control lateral COM motion during walking to improve functional balance and gait in people with iSCI. The clinical interventions could focus more on traditional balance training tasks, like narrow base of support walking, rapid maneuvers during walking, or manual perturbations to improve lateral balance control. Alternately, a novel approach to train lateral COM control in people with iSCI could be to perform gait training in a movement amplification field that applies proportional forces in the same direction as the real-time lateral velocity of the participant (63, 64).

There are a few limitations associated with this study. While our findings suggest that lateral COM motion during walking is associated with clinical gait and balance outcomes, it is not clear if this relationship is causative. Future research is recommended to investigate if there is a causal relationship between these measures. Our current study has a small sample size and relatively high functioning (community dwelling), ambulatory people with iSCI were included. Thus, findings from our study cannot be generalized to all individuals with iSCI but should be important for those who walk with limited or no assistive devices.

## Conclusion

This study finds that the ability to control lateral COM motion during walking correlates with previously validated clinical gait and balance measures and may be a contributing factor to gait and balance outcomes in people with iSCI. Further research should explore if interventions designed to improve control of lateral COM motion during walking translate to improvements in functional gait and balance in people with iSCI.

## Data availability statement

The raw data supporting the conclusions of this article will be made available by the authors, without undue reservation.

## Ethics statement

The studies involving human participants were reviewed and approved by Institutional Review Boards at Northwestern University and the Edward Hines Jr. Veterans Affairs Hospital. The patients/participants provided their written informed consent to participate in this study. Written informed consent was obtained from the individual(s) for the publication of any potentially identifiable images or data included in this article.

## Author contributions

KG, WO, AS, TC, and SD designed the study. KG, AS, HH, and SD collected the data. AS, SD, K-YK, and KG analyzed the data. All authors contributed to the article and approved the submitted version.

## Funding

This study was funded by the United States Department of Veteran Affairs, Office of Rehabilitation Research & Development, #1 I01 RX003371.

## Conflict of interest

The authors declare that the research was conducted in the absence of any commercial or financial relationships that could be construed as a potential conflict of interest.

## Publisher’s note

All claims expressed in this article are solely those of the authors and do not necessarily represent those of their affiliated organizations, or those of the publisher, the editors and the reviewers. Any product that may be evaluated in this article, or claim that may be made by its manufacturer, is not guaranteed or endorsed by the publisher.

## Supplementary material

SUPPLEMENTARY VIDEO The video demonstrates the treadmill-based task used to evaluate participant’s capacity to control their lateral COM motion during walking. The video shows a participant walking on an oversized treadmill under the supervision of two physical therapists. The participant receives visual feedback of their real-time mediolateral COM position, represented by a white line projected along the length of the treadmill, and a target lane, represented by two green lines. The participant is instructed to try and maintain the white line within the target lane. During the task, if the participant’s COM moves outside the target lane the area outside the target turns red providing the participant with an immediate visual cue to try and move their COM within the target lane. To challenge each participant’s capacity to control their lateral COM motion, the target lane progressively narrows or widens based on their performance.
